# Additive relationship between serum fibroblast growth factor 21 level and coronary artery disease

**DOI:** 10.1186/1475-2840-12-124

**Published:** 2013-08-28

**Authors:** Yun Shen, Xiaojing Ma, Jian Zhou, Xiaoping Pan, Yaping Hao, Mi Zhou, Zhigang Lu, Meifang Gao, Yuqian Bao, Weiping Jia

**Affiliations:** 1 Department of Endocrinology and Metabolism, Shanghai Jiao Tong University Affiliated Sixth People’s Hospital, Shanghai Key Laboratory of Diabetes Mellitus, Shanghai Diabetes Institute, Shanghai, China; 2 Department of Cardiology, Shanghai Jiao Tong University Affiliated Sixth People’s Hospital, Shanghai, China

**Keywords:** Fibroblast growth factor 21, Coronary artery disease, Non-alcoholic fatty liver disease, Metabolic syndrome

## Abstract

**Background:**

Expression and activity of the fibroblast growth factor (FGF) 21 hormone-like protein are associated with development of several metabolic disorders. This study was designed to investigate whether serum FGF21 level was also associated with the metabolic syndrome-related cardiovascular disease, atherosclerosis, and its clinical features in a Chinese cohort.

**Methods:**

Two-hundred-and-fifty-three subjects visiting the Cardiology Department (Sixth People's Hospital affiliated to Shanghai JiaoTong University) were examined by coronary arteriography (to diagnose coronary artery disease (CAD)) and hepatic ultrasonography (to diagnose non-alcoholic fatty liver disease (NAFLD)). Serum FGF21 level was measured by enzyme-linked immunosorbent assay and analyzed for correlation to subject and clinical characteristics. The independent factors of CAD were determined by multivariate logistic regression analysis.

**Results:**

Subjects with NAFLD showed significantly higher serum FGF21 than those without NAFLD (388.0 pg/mL (253.0-655.4) vs. 273.3 pg/mL (164.9-383.7), *P* < 0.01). Subjects with CAD showed significantly higher serum FGF21, regardless of NAFLD diagnosis (*P* < 0.05). Serum FGF21 level significantly elevated with the increasing number of metabolic disorders (*P* for trend < 0.01). After adjustment of age, sex, and BMI, FGF21 was positively correlated with total cholesterol (*P* < 0.05) and triglyceride (*P* < 0.01). FGF21 was identified as an independent factor of CAD (odds ratio = 2.984, 95% confidence interval: 1.014-8.786, *P* < 0.05).

**Conclusions:**

Increased level of serum FGF21 is associated with NAFLD, metabolic disorders and CAD.

## Introduction

The circulating hormone-like fibroblast growth factor (FGF) 21 is primarily synthesized by the liver (and to a lesser extent by the skeletal muscle and pancreas), but plays an important systemic role in regulating glucolipid metabolism and insulin sensitivity [1], [2]. The mechanism of FGF21 activity includes stimulation of glucose transporter protein-1 (GLUT-1) expression to promote glucose uptake and metabolism by fat cells and long-term energy storage [3]. In addition, FGF21 inhibits hepatic glycogen degradation, thereby reducing levels of circulating glugagon and helping to maintain the tight hormonal balance required for proper physiology [4]. All of these FGF21-mediated effects culminate in a reduction of blood glucose levels and maintenance of the non-diabetic state.

A key feature of diabetes is perturbed lipid metabolism, and FGF21 has been implicated in this systemic physiological response as well. Specifically, intravenous injection of FGF21 in rats was shown to cause marked decreases in serum levels of triglyceride (TG), free fatty acid, and total cholesterol (TC) [5]. Large epidemiological studies of human populations have shown that significantly enhanced levels of FGF21 accompany obesity, impaired glucose tolerance, type 2 diabetes, and non-alcoholic fatty liver disease (NAFLD) [2], [6], [7].

Perturbed glucolipid metabolism, and the metabolic syndrome-NAFLD combination are well known risk factors for coronary artery disease (CAD) [8]. The associations of FGF21 and these important risk factors of CAD prompted our current investigation of whether a direct relationship exists between serum FGF21 levels and CAD occurrence in the presence or absence of concomitant NAFLD.

## Materials and methods

### Study participants

Subjects visiting the Cardiology Department of the Sixth People's Hospital Affiliated to Shanghai Jiao Tong University (China) for examination by coronary arteriography were recruited for this study between July 2008 and January 2010. Study enrollment was denied according to the presence of acute or chronic viral hepatitis, drug-induced or alcoholic liver diseases, alcoholism (a total of ≥ 140 g per week by a male adult or a total of ≥ 70 g per week by a female [9]), total parenteral nutrition, chronic kidney diseases, hyper- or hypothyroidism, cancer, and current treatment with systemic corticosteroids; furthermore, subjects with incomplete anthropometric or laboratory data were excluded from analysis.

The study was carried out with pre-approval from the local Ethics Committee of the Sixth People's Hospital, and all enrolled subjects provided informed consent.

### Physical examination and laboratory testing

The body mass index (BMI) was calculated as weight (in kilograms) divided by squared-height (in meters). Blood pressure (BP) was measured by a sphygmomanometer. Waist circumference (WC) was measured by around the abdomen, starting horizontally at the midpoint of the costal margin and following the iliac crest on the mid-axillary line. Fasting plasma glucose (FPG; measured after a 10 h overnight fast) and 2 h-postprandial plasma glucose (2hPG) were assessed by the glucose oxidase method. Glycated hemoglobin A1c (HbA1c) was detected by high-pressure liquid chromatography (Variant II; Bio-Rad, Hercules, CA, USA). Serum lipid profiles including TG, TC, low-density lipoprotein cholesterol (LDL-c) and high-density lipoprotein cholesterol (HDL-c) were detected via standard enzymatic procedures on the 7600–020 autoanalyzer (Hitachi, Tokyo, Japan). Serum C-reactive protein (CRP) was measured by particle-enhanced immunonephelometry assay (Dade Behring Inc., Newark, NJ, USA). Fasting serum insulin was assayed by radioimmunoassay (Linco Research, St. Charles, MO, USA) and used to calculate the Homeostasis Model of Assessment - Insulin Resistance (HOMA-IR) as follows: [FPG (mmol/L) × fasting serum insulin (mU/L)] / 22.5. Serum FGF21 was detected by enzyme-linked immunosorbent assay (Antibody and Immunoassay Services, University of Hong Kong), which gave inter-batch and intra-batch variances of 7.8% and 9.1%, respectively.

### Coronary arteriography and CAD diagnosis

The coronary arteriography was carried out using standard Judkins technique [10], and all major coronary arteries were examined in at least two orthogonal views. The arteriographic analysis was conducted by two experienced interventional cardiologists, who were blinded to the study’s objective and design. CAD was diagnosed when stenosis was detected in ≥50% of the lumen diameter of a major coronary artery, including the left main coronary artery, left anterior descending artery or its first diagonal branch, left circumflex artery or its first obtuse marginal branch, and right coronary artery.

### Diagnostic criteria for metabolic disorders and NAFLD

Metabolic syndrome (MS) and its components were defined according to the 2007 Joint Committee for Developing Chinese Guidelines (JCDCG2007) [11], by presence of ≥ 3 of the following risk factors: abdominal obesity (defined as WC > 90 cm for men and > 85 cm for women); serum TG level ≥ 1.7 mmol/L or specific treatment for lipid abnormality; serum HDL-c level < 1.04 mmol/L or specific treatment for lipid abnormality; BP ≥ 130/85 mmHg or treatment of previously diagnosed hypertension; FPG of ≥ 6.1 mmol/L and/or 2hPG of ≥ 7.8 mmol/L or previously diagnosed type 2 diabetes. Dyslipidemia was defined as the margin rising of serum lipid level according to the JCDCG defintion, including serum TC level ≥ 5.18 mmol/L; serum TG level ≥ 1.7 mmol/L; serum LDL-c level ≥ 3.37 mmol/L; serum HDL-c level < 1.04 mmol/L; or specific treatment for dyslipidemia [11]. NAFLD was diagnosed by B ultrasonography. Hepatic steatosis was defined by a diffuse increase of fine echoes in the liver parenchyma compared with that in the kidney or spleen parenchyma, according to the 2010 Prevention and Treatment Guidelines for NAFLD published by the Society of Hepatology, Chinese Medical Association [9]. Smoking status was defined as at least one cigarette per day the past six months or more [12].

### Statistical analysis

The SPSS software suite, version 19.0, was used for all statistical analyses. Normally distributed data were expressed as mean ± standard deviation, and skewed data were expressed as median (inter-quartile range). Inter-group comparisons of clinical values were carried out by the unpaired student’s t test (normally distributed data) or the Mann–Whitney U test (skewed data). Inter-group comparisons of categorical variables were carried out by the chi-square test. Partial correlation analysis was performed to explore the relationship between FGF21 and clinical parameters. Multivariate logistic regression analysis was performed to identify the independent factors of CAD occurrence. All *P*-values were two-tailed and considered statistically significant at < 0.05.

## Results

### Subject characteristics

The study was composed of 253 subjects, represented by 164 men and 89 women, between the ages of 38 and 86 years-old (mean age: 66.3 ± 10.1). All women were postmenopausal. Serum FGF21 level of men and women was 291.7 pg/ml (194.0-411.8) and 319.2 pg/ml (198.3-568.8) respectively with a non-significant result of comparison (*P* = 0.317). Abdominal ultrasonography diagnosed 183 of the subjects without NAFLD. Subjects with NAFLD showed a significantly higher level of serum FGF21 (388.0 pg/mL (253.0-655.4) vs. those without NAFLD: 273.3 pg/mL (164.9-383.7), *P* < 0.01).

The NAFLD and non-NAFLD groups were further divided into two subgroups according to the presence of CAD judged by the coronary arteriography. As shown in Table 1, among subjects without NAFLD, those with CAD showed significantly higher age, 2hPG, and significantly lower HDL-c and TC (vs. subjects without CAD, all *P* < 0.05). While among subjects with NAFLD, those with CAD and without CAD showed significant differences also in serum TG besides serum FGF21 (*P* < 0.05). Concerning the components of MS, only the frequency of hypertriglyceridemia was higher in CAD subjects with NAFLD (vs. non-CAD subjects with NAFLD, *P* < 0.01).

**Table 1:** Subject characteristics

	**Without NAFLD**			**With NAFLD**		
**Variables**	**Total**	**Non-CAD**	**CAD**	**Total**	**Non-CAD**	**CAD**
	**N = 183**	**N = 47**	**N = 136**	**N = 70**	**N = 27**	**N = 43**
Sex(Male/Female)	121/62	23/24	98/38	43/27	16/11	27/16
Age, years	67.2 ± 10.0	64.6 ± 9.9	68.2 ± 9.9^*^	63.9 ± 9.9^#^	61.3 ± 7.7	65.5 ± 10.9
Body mass index, kg/m^2^	23.9 ± 3.1	24.0 ± 3.5	23.9 ± 2.9	26.5 ± 3.7^##^	26.5 ± 4.9	26.5 ± 2.8
Waist circumference, cm	88.4 ± 9.3	86.7 ± 9.8	88.9 ± 9.0	95.4 ± 9.3^##^	96.6 ± 11.7	94.7 ± 7.6
Systolic blood pressure, mmHg	130.0	130.0	130.0	140.0	140.0	140.0
	(120.0-148.0)	(119.0-150.0)	(120.0-147.8)	(128.8-151.5)^#^	(120.0-150.0)	(130.0-153.0)
Diastolic blood pressure, mmHg	80.0	80.0	80.0	80.0	80.0	80.0
	(70.0-80.0)	(70.0-85.0)	(70.0-80.0)	(71.8-90.0)^##^	(70.0-85.0)	(72.0-90.0)
Fasting plasma glucose, mmol/L	5.3(4.9-6.1)	5.3(5.0-5.9)	5.3(4.9-6.1)	5.8(5.2-7.2)^##^	5.6(5.1-6.6)	5.9(5.4-7.7)
2 h postprandial glucose, mmol/L	7.9(6.4-10.7)	7.5(6.1-9.3)	8.0(6.5-11.5)^*^	10.4(8.2-12.9)^##^	9.7(7.9-12.9)	11.3(8.2-12.9)
HbA1c,%	6.1(5.7-6.5)	5.9(5.6-6.3)	6.2(5.8-6.6)	6.5(5.9-7.6)^##^	6.3(5.7-7.1)	6.6(6.2-7.9)
HOMA-IR	3.8(2.4-5.3)	3.7(2.4-5.4)	3.9(2.4-5.3)	6.0(3.7-7.2)^##^	5.9(3.7-6.4)	6.6(3.7-9.2)
Total cholesterol, mmol/L	4.3 ± 1.0	4.6 ± 1.1	4.2 ± 1.0^*^	4.7 ± 1.3^#^	4.5 ± 1.4	4.8 ± 1.2
Triglyceride, mmol/L	1.4(1.0-1.9)	1.4(0.9-2.0)	1.4(1.0-1.8)	2.2(1.4-3.1)^##^	1.7(1.3-2.4)	2.6(1.6-3.7)^*^
HDL-c, mmol/L	1.1 ± 0.3	1.2 ± 0.3	1.1 ± 0.3^*^	1.0 ± 0.3^##^	1.0 ± 0.3	1.0 ± 0.3
LDL-c, mmol/L	2.9 ± 1.0	3.0 ± 0.9	2.9 ± 1.0	3.1 ± 1.0	3.1 ± 1.0	3.2 ± 1.0
C-reactive protein, mg/L	1.2(0.5-3.1)	1.0(0.4-3.0)	1.2(0.5-3.3)	2.3(1.0-6.9)^##^	1.6(0.7-6.3)	2.5(1.1-7.6)
FGF21, pg/mL	273.3	256.1	277.8	388.0	321.5	415.5
	(164.9-383.7)	(150.6-351.9)	(171.1-406.6)^*^	(253.0-655.4)^##^	(239.1-497.6)	(258.0-693.7)^*^
Smoker n (%)	82(44.8)	16(34.0)	66(48.5)	31(44.3)	12(44.4)	19(44.2)
Metabolic syndrome and its components						
Abdominal obesity n (%)	93(50.8)	22(46.8)	71(52.2)	55(78.6)^##^	21(77.8)	34(79.1)
Hypertriglyceridemia n (%)	87(47.5)	20(42.6)	67(49.3)	50(71.4)^##^	14(51.9)	36(83.7)^**^
Low HDL-c n (%)	73(39.9)	15(31.9)	58(42.6)	41(58.6)^#^	14(51.9)	27(62.8)
Hypertension n (%)	160(87.4)	41(87.2)	119(87.5)	63(90.0)	23(85.2)	40(93.0)
Hyperglycemia n (%)	105(57.4)	25(53.2)	80(58.8)	56(80.0)^##^	21(77.8)	35(81.4)
Metabolic syndrome n (%)	105(57.4)	24(51.1)	81(59.6)	63(90.0)^##^	23(85.2)	40(93.0)
Dyslipidemia n (%)	142(77.6)	34(72.3)	108(79.4)	68(97.1)^##^	25(92.6)	43(100.0)

Data were expressed as mean ± SD or median (interquartile range). For both the non-NAFLD and NAFLD groups: ^*^P < 0.05 CAD vs. non-CAD; ^**^P < 0.01 CAD vs. non-CAD. For the total subjects with and without NAFLD: ^#^*P* < 0.05 NAFLD vs. non-NAFLD; ^##^*P* < 0.01 NAFLD vs. non-NAFLD.

**Figure 1:**
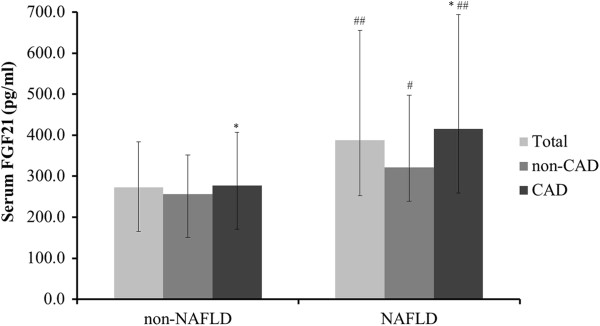
**Differential serum FGF21 level among subjects with NAFLD and/or CAD.** White bars, total; grey bars, non-CAD subgroup; black bars, CAD subgroup. ^*^P < 0.05, CAD subgroup vs. non-CAD subgroup; ^#^*P* < 0.05, ^##^*P* < 0.01, NAFLD group vs*.* non-NAFLD group.

As seen in Figure 1, both subjects with and without CAD showed a significant elevation of serum FGF21 level in the NAFLD group compared to the non-NAFLD group (415.5 pg/mL (258.0-693.7) vs*.* 277.8 pg/mL (171.1-406.6), *P* < 0.01; 321.5 pg/mL (239.1-497.6) vs. 256.1 pg/mL (150.6-351.9), *P* < 0.05). Similarly in subjects with and without NAFLD, serum FGF21 level was elevated in the CAD subgroup compared to the non-CAD subgroup (415.5 pg/mL (258.0-693.7) vs*.* 321.5 pg/mL (239.1-497.6), *P* < 0.05; 277.8 pg/mL (171.1-406.6) vs. 256.1 pg/mL (150.6-351.9), *P* < 0.05).

### Association of serum FGF21 level with MS and -related clinical parameters

**Figure 2:**
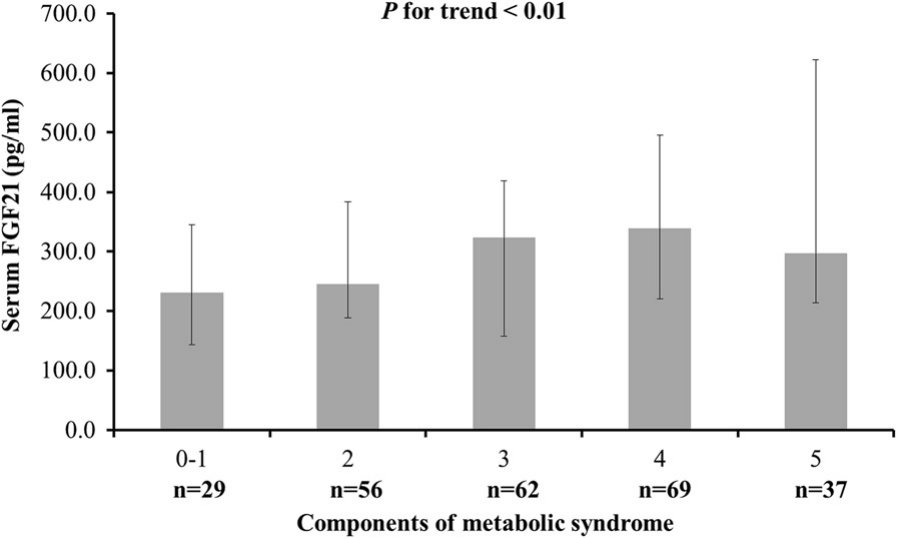
**Differential serum FGF21 level among subjects with various components of MS.***P* < 0.01 for trend of elevated serum FGF21 level with increasing number of disorders.

When the subjects were grouped according to MS status and divided into subgroups according to the particular components of MS, a trend in increasing serum FGF21 level accompanying increased numbers of MS-related disorders was noticed (*P* for trend < 0.01; Figure 2). After adjustment of age, sex and BMI, correlation analysis showed that a significant positive relationship existed between FGF21 and TC (*P* < 0.05), as well as TG (*P* < 0.01; Table 2).

**Table 2:** Partial correlation analysis between FGF21 and clinical variables

	**Partial correlation**	
**Variables**	**R**	***P***
Waist circumference	0.102	0.129
Systolic blood pressure	0.100	0.136
Diastolic blood pressure	0.040	0.548
Fasting plasma glucose	0.077	0.248
2 h postprandial glucose	0.056	0.405
HbA1c	0.027	0.684
HOMA-IR	0.080	0.233
Total cholesterol	0.154	0.021
Triglyceride	0.317	<0.001
High-density lipoprotein cholesterol	−0.095	0.157
Low-density lipoprotein cholesterol	0.077	0.248
C-reactive protein	0.068	0.314

Partial correlation was made after adjustment for age, sex, and BMI.

### Association between serum FGF21 level and CAD

Multivariate logistic regression analysis was structured with independent variables of age, sex, BMI, WC, HOMA-IR, FGF21, smoking status, hyperglycemia, family history of CAD, hypertension, NAFLD, and dyslipidemia (Table 3). Consequently, male subjects were found more prone to CAD (*P* < 0.01). In addition, serum FGF21 level was identified as one of the independent factors of CAD, along with age. The odds ratio of FGF21 was 2.984 with a 95% confidence interval of 1.014-8.786 (*P* < 0.05).

**Table 3:** Independent factors of CAD identified by multivariate logistic regression analysis

	**β**	**S.E**	**OR**	***P***
Sex	−0.882	0.304	0.414(0.228-0.752)	0.004
FGF21^a^	1.093	0.551	2.984(1.014-8.786)	0.047
Age	0.043	0.015	1.044(1.013-1.076)	0.005

Variables included in the model were age, sex, BMI, WC, HOMA-IR, FGF21, smoking status, hyperglycemia, family history of CAD, hypertension, NAFLD, and dyslipidemia.

^a^Data were after logarithm.

## Discussion

The contribution of metabolic disorders to development and progression of cardiovascular diseases has been extensively studied. In recent years, however, the important role of cytokines, and resultant inflammation, in this etiological relationship has been recognized. Mainly characterized as a metabolic factor, the newly-recognized cytokine characteristics of FGF21 have stimulated research interest in its putative roles in the inflammation-related processes of metabolic disorders and diseases. The collective evidence, however, has revealed a more complex role for this factor in normal metabolic processes and pathogeneses. For example, animal studies have demonstrated that FGF21 promotes glycolipid metabolism and insulin sensitivity but clinical studies of humans have yielded opposite results [13], [14]. Mraz M, et al. reported that serum FGF21 level becomes elevated under conditions of insulin resistance [15], and An et al. showed that increased serum FGF21 level accompanied the development of carotid artery plaques in patients with type 2 diabetes [16].

Studies of the relationship of enhanced FGF21 levels with NAFLD have provided slightly more consistent evidence. Cross-sectional clinical studies have demonstrated an incremental increase in FGF21 levels with increased severity of NAFLD [2]; in particular increased mRNA expression of FGF21 has been shown in hepatic biopsies [6]. Furthermore, a 3-year follow-up of NAFLD subject outcome indicated that serum FGF21 level might be a clinically-relevant disease biomarker, suggesting its potential for monitoring response to therapy [17]. In the present study, NAFLD subjects were found to have higher level of serum FGF21 and those subjects with multiple metabolic disorders were found to have the highest level of serum FGF21, thereby supporting the hypothesis that this factor is involved in glycolipid metabolism.

The etiological process leading from NAFLD to atherosclerosis is believed to involve a cluster of metabolic disorders and other co-morbidities (possibly inflammation-related). The most dangerous atherosclerosis manifestation is coronary atherosclerotic heart disease and research efforts have focused on defining the relationship between metabolic-related and inflammation-related serum factors (such as FGF21) and CAD. In a recent study to determine the clinical profile of CAD, using findings from electrocardiogram, serology and physical symptoms (such as chest discomfort), it was found that elevated serum FGF21 level was a distinctive marker [18]; this observation also served to indicate that FGF21 might be involved in the pathophysiological process of CAD. Lee et al. studied the relation between serum FGF21 and CAD diagnosis according to computed tomography findings and found that serum FGF21 level was significantly correlated with serum TG, LDL-c, HOMA-IR and the occurrence of MS; however, no relationship was found between serum FGF21 level and CAD diagnosed by computed tomography [19]. Both of these studies of the clinical features of CAD relied on in-patient populations with a high risk of various metabolic disorders, but ignored the possible influence of NAFLD. In the current study of the relationship between FGF21 and CAD, subjects undergoing coronary arteriography were analyzed. A significant elevation of serum FGF21 among CAD subjects was discovered independently of NAFLD status. Multivariate logistic regression analysis also identified serum FGF21 level as one of the independent factors of CAD occurrence.

Enhanced serum FGF21 level has been previously demonstrated in subjects with obesity, diabetes, and dyslipidemia. Moreover, the enhanced serum FGF21 level has been correlated to presence of insulin resistance, and increased levels of TC and TG. For example, Cheng et al. identified FGF21 as a predictive marker of diabetes, but also showed that the elevated level did not correlate with disease duration [20]. Similarly, Li et al. reported significantly increased level of FGF21 in individuals with impaired glucose tolerance, and showed a significant positive relationship of FGF21 level with TC and TG level [21]. The findings of the current study presented herein agree with these data, collectively supporting the hypothesis that serum FGF21 level is likely involved in the process of CAD; the current study provides novel insights into the contribution of FGF21 being independent of such traditional cardiac risk factors as age, hyperglycemia, hypertension, dyslipidemia, and even NAFLD.

Insulin resistance is a main pathophysiological foundation of NAFLD, along with type 2 diabetes and even MS and cardiovascular diseases. Serum FGF21 level has been found to be higher in insulin resistance status in both human-based studies and rat experiments [22]. In addition, Shargorodsky M, et al. found that subjects with significant improvement of insulin resistance also tended to show significant improvement on central aortic augmentation index. This result suggested that insulin resistance might be involved in the process of arterial lesions [23]. A Japanese study even recommended using ALT/AST ratio to reflect the extent of insulin resistance [24]. Therefore, FGF21, a mainly liver-derived cytokine, was found to be associated with the occurrence of CAD in this study, which might be explained, concerning the mechanism, by the involvement of insulin resistance.

Individuals with a cluster of metabolic disorders have increased serum FGF21 level, and research has implicated the up-regulation of this cytokine as acting to compensate for the abnormal metabolic status [25]. This compensatory mechanism may explain the apparent inconsistent findings from animal and human studies of FGF21 and metabolic disorders. Elevated mRNA expression of FGF21 was found in rat cardiac micro-vascular endothelial cells (CMECs) cultured in atherosclerosis-like conditions [26]; furthermore, exogenous FGF21 infusion to the CMEC atherosclerosis-promoting culture significantly inhibited the cells’ apoptosis. These findings suggested that up-regulated FGF21 expression might be protective at the early stage of atherosclerosis, helping the cells to recover normal endothelial function. Thus, it is possibly that the elevated FGF21 observed in the CAD subjects of our study represent a similar compensatory mechanism, by which the system is attempting to protect against atherosclerosis. While this study is the first to provide clinical evidence of the relationship between serum FGF21 level and CAD diagnosed by coronary arteriography, further animal studies should be conducted to reveal the exact mechanism between FGF21 and atherosclerosis.

### Limitations

Some inherent features of the current study’s design may have affected the results and may limit generalization of the findings. First, the sample size was relatively small for the cross-sectional study design and some inherent bias may have been masked. Second, the study population was relatively homogenous, characterized by Chinese adults, middle- and old-aged, presenting at a single health institute and focused clinical care department (cardiology); generally, these subjects represent a high-risk of CAD.

## Conclusions

In a study of subjects undergoing coronary angiography, serum FGF21 level was significantly higher among the individuals with NAFLD. Moreover, the elevated serum FGF21 level followed a positive incremental trend in conjunction with amount of metabolic disorders present in the patient. Finally, subjects with CAD had higher serum FGF21 level than those without CAD, regardless of NAFLD status, and FGF21 was identified as an independent factor of CAD. Considering these results in conjunction with the collective findings from previous related clinical studies, serum FGF21 elevation may represent a regulatory compensation mechanism under atherosclerosis conditions, and may represent a promising therapeutic target for this disease.

## Abbreviations

BMI: Body mass index; BP: Blood pressure; CAD: Coronary artery disease; CRP: C-reactive protein; FGF: Fibroblast growth factor; FPG: Fasting plasma glucose; GLUT-1: Glucose transporter protein-1; HDL-c: High-density lipoprotein cholesterol; HOMA-IR: Homeostasis model assessment-insulin resistance; LDL-c: Low-density lipoprotein cholesterol; MS: Metabolic syndrome; NAFLD: Non-alcoholic fatty liver disease; TC: Total cholesterol; TG: Triglyceride; WC: Waist circumference; 2hPG: 2 h postprandial plasma glucose.

## Competing interests

All authors declare that they have no competing interests.

## Authors’ contributions

YB and WJ designed the study. MZ, YH, and MG collected data. YS analyzed data and wrote the draft. XP measured FGF21. JZ and ZL did the angiographic analysis. XM, YB, and WJ revised the paper and contributed to the discussion. All authors read and approved the final manuscript.
